# The association of women’s birth size with risk of molecular breast cancer subtypes: a cohort study

**DOI:** 10.1186/s12885-021-08027-9

**Published:** 2021-03-23

**Authors:** Marie S. Sandvei, Signe Opdahl, Marit Valla, Pagona Lagiou, Ellen Veronika Vesterfjell, Tor Vikan Rise, Tina Syvertsen Overrein, Anette H. Skjervold, Monica J. Engstrøm, Anna M. Bofin, Lars J. Vatten

**Affiliations:** 1 Faculty of Medicine and Health Sciences, Department of Public Health and Nursing, Norwegian University of Science and Technology, Post box 8905, N-7491 Trondheim, Norway; 2 The Cancer Clinic, St. Olavs Hospital, Trondheim University Hospital, Trondheim, Norway; 3 NTNU, Faculty of Medicine and Health Sciences, Department of Clinical and Molecular Medicine, Faculty of Medicine and Health Sciences, Norwegian University of Science and Technology, Post box 8905, N-7491 Trondheim, Norway; 4 Department of Pathology, St. Olavs Hospital HF, Trondheim University Hospital, Postboks 3250 Torgarden, 7006 Trondheim, Norway; 5 Department of Hygiene, Epidemiology and Medical Statistics, School of Medicine, National and Kapodistrian University of Athens, 75 M. Asias Street, Goudi, GR-115 27 Athens, Greece; 6 Department of Epidemiology, Harvard School of Public Health, Boston, USA; 7 Department of Breast and Endocrine Surgery, St. Olavs Hospital, Trondheim University Hospital, Trondheim, Norway

**Keywords:** Breast cancer, Molecular subtypes, Epidemiology, Risk factors, Birth size, Birth length, Birth weight

## Abstract

**Background:**

Because birth size appears to be positively associated with breast cancer risk, we have studied whether this risk may differ according to molecular breast cancer subtypes.

**Methods:**

A cohort of 22,931 women born 1920–1966 were followed up for breast cancer occurrence from 1961 to 2012, and 870 were diagnosed during follow-up. Archival diagnostic material from 537 patients was available to determine molecular breast cancer subtype, specified as Luminal A, Luminal B (human epidermal growth factor receptor 2 (HER2)-), Luminal B (HER2+), HER2 type, and Triple negative (TN) breast cancer. Information on the women’s birth weight, birth length and head circumference at birth was used to estimate hazard ratios (HR) with 95% confidence intervals (CI) for each molecular subtype, applying Cox regression, and stratified by maternal height.

**Results:**

Birth length (per 2 cm increments) was positively associated with Luminal A (HR = 1.2, 95% CI, 1.0–1.3), Luminal B (HER2+) (HR = 1.3, 95% CI, 1.0–1.7), and TN breast cancer (HR = 1.4, 95% CI, 1.0–1.9). No clear association was found for birth weight and head circumference. The positive associations of birth length were restricted to women whose mothers were relatively tall (above population median).

**Conclusion:**

We found a positive association of birth length with risk of Luminal A, Luminal B (HER2+) and TN breast cancer that appears to be restricted to women whose mothers were relatively tall. This may support the hypothesis that breast cancer risk is influenced by determinants of longitudinal growth and that this finding deserves further scrutiny.

## Background

It has been demonstrated that breast cancer can be classified into different molecular subtypes using gene expression analysis [1]. However, molecular subtyping can also be achieved using surrogate markers for gene expression analysis [2–4]. Thus, using immumohistochemistry for detection of protein expression and in situ hybridisation for assessment of gene copy number, tumours can be classified into different subtypes. The differences between subtypes underline the heterogeneous nature of breast cancer, including different pathogeneses [5] and clinical course [1, 2].

Therefore, it is plausible that underlying risk factors may also differ between subtypes [6–9], indicating a need for subtype specific revision of breast cancer epidemiology, including possible consequences for prevention strategies. Recent results suggest that the reduced risk associated with early age at first birth, high parity and overweight before menopause, may also vary according to subtype [6–9],

Until recently, factors associated with birth size, i.e. birth weight, birth length and head circumference, have not been comprehensively studied in relation to risk of molecular subtypes of breast cancer. However, the association of birth size has been assessed in relation to hormone receptor status of the tumours; thus, there is some evidence for a positive association of birth weight with risk for ER positive breast cancer, but not for ER negative tumours [10].

The hypothesis that breast cancer may originate in utero is based on the fact that the mammary gland, which is not fully developed at birth, is exposed to high levels of intrauterine mammotropic hormones [11, 12]. This may increase the mammary gland-specific stem cell pool and eventually, the likelihood for cancer initiation later in life [13]. This hypothesis is supported by different lines of research [14–20] that combined may suggest a coherent sequence of plausible events. Thus, cord blood hormones could partly determine the size of the mammary stem cell pool and simultaneously influence fetal growth. Therefore, birth size may be positively associated with breast cancer risk later in life [17].

In this follow-up study, we used perinatal data from a cohort of Norwegian women born at one particular hospital between 1920 and 1966. During follow-up from 1961 to 2012, we studied women who were diagnosed with breast cancer at St. Olavs Hospital in Trondheim, Norway, and used surrogate markers applied to tissue microarrays of archival tumour tissue to determine the following molecular subtypes: Luminal A, Luminal B (HER2-), Luminal B (HER2+), and the non-luminal subtypes, the HER2 type and TN breast cancer. Our aim was to assess whether indicators of birth size are differentially associated with the risk for luminal and non-luminal subtypes.

## Methods

A unique ID number was assigned to all Norwegian residents who were alive in 1960, and is assigned to all new residents from 1961 onwards. Each resident’s record is continuously updated on vital status and residential and childbearing history through the National Registry. By combining the mother’s name and the daughter’s unique 11-digit number, we could identify women who were born at St. Olavs hospital, Trondheim University Hospital, between 1920 and 1966. After excluding 524 twins, 111 triplets, 32 women with missing information on plurality, and 12 women whose identity could not be determined with certainty, 22,931 women born between 1920 and 1966 were eligible for breast cancer follow-up until the end of 2012.

The reporting of cancer to the Cancer Registry of Norway is mandatory, and breast cancer is registered according to the international classification of diseases (ICD-7, code 170). For women born before 1941, breast cancer follow-up started on January 1st, 1961, and for women born after 1940, follow-up started at their 20th birthday. Follow-up ended when a cancer (at any site) was diagnosed, at emigration or at death (from any cause), or on December 31st, 2012, whichever occurred first. During follow-up, a total of 870 women were diagnosed with incident breast cancer. Among them, 598 were diagnosed at St Olav’s Hospital; archival diagnostic tissue was available for all these 598 patients, and molecular subtyping proved to be successful for 537 of these cases.

From the birth record of each of the 22,931 women, we extracted information on birth weight (g), birth length (cm), head circumference (cm), gestational age (in weeks or months), and birth order. We also extracted maternal information, including maternal age at childbearing, height, marital status, and socioeconomic status (occupation). (Baseline characteristics of the cohort members according to birth size are shown in Table 1).

**Table 1:** Baseline characteristics of cohort members, follow-up of 22,931 women

	Number (%)
Mother’s age at birth, mean (SD)	28.0 (6.1)
Birth weight, g	
Mean (SD)	3446 (508)
< 3000 g	3684 (16.1)
3000–3499 g	8657 (37.8)
3500–3999 g	7672 (33.5)
≥ 4000 g	2912 (12.7)
Missing	6 (0.03)
Birth length, cm	
Mean (SD)	50.5 (2.1)
< 49	3190 (13.9)
49	3055 (13.3)
50	5298 (23.1)
51	4317 (18.8)
≥ 52	7066 (30.8)
Missing	5 (0.02)
Head circumference, cm	
Mean (SD)	34.8 (1.5)
< 34	3849 (16.8)
34	5397 (23.5)
35	6408 (27.9)
36	4642 (20.2)
≥ 37	2549 (11.1)
Missing	86 (0.4)
Total	22,931 (100)

From the National Registry, we collected information on childbearing history in adulthood for cohort members who were born in 1930 or later, and who were still residents in Trondheim. Thus, information on age at first birth and parity, both of which are associated with breast cancer risk, were included as co-variables for a subset (78%) of the population.

### Molecular subtypes of breast cancer using archived diagnostic tissue

Prior to molecular subtyping, each case was re-examined by two independent pathologists and classified into histological type and grade according to current guidelines. Sections from representative paraffin tumour blocks were stained with hematoxylin–erythrosine–saffron (HES), and classified into histopathologic type and grade. Tumour size was correlated to information in the pathology report. Tissue microarrays (TMA) were constructed as previously described and referenced in detail by Engstrøm et al. (4) and Valla et al. (3).

Briefly, immuno-staining was performed for ER, PR, HER2, the proliferation marker Ki67, and Basal markers and EGFR. Negative controls were included in all staining runs. ER and PR were positive when ≥1% of tumor nuclei showed positive staining and Ki67 was counted in 500 tumor cells (hotspots), and considered high when ≥15% of nuclei were positive, both irrespective of staining intensity. Membranous staining for HER2 was scored from 0 to + 3, and HER2 amplification was defined as a gene to chromosome ratio ≥ 2.

For CK5 and EGFR, a staining index was calculated by multiplying the proportion of positive staining cells [1 (< 10%); 2 (10–50%); 3 (> 50%)] by staining pattern/intensity. Staining intensity for CK5 was defined as 0 (no staining); 1 (weak); 2 (moderate), and 3 (strong). For EGFR, membranous staining was 0 (no staining); 1 (faint, incomplete staining); 2 (moderate intensity, circumferential staining); 3 (strong intensity, circumferential staining), according to Dako PharmDX Kit guidelines. A staining index of 0–1 was classified as negative, and 2–9 as positive.

Breast cancers were then reclassified into molecular subtypes by immunohistochemistry and fluorescence in situ hybridization, using the surrogate markers for oestrogen receptor (ER), progesterone receptor (PR), and the proliferation markers Ki67 and HER2. In addition, Cytokeratin 5 and Epidermal growth factor receptor 1 were used to differentiate between the Basal phenotype and the Five negative phenotype, however, we combined these subtypes into one, notified as TN.

ER was positive when ≥1% of tumour cells showed positive nuclear staining, and ≥ 10% for PR to be positive [21]. The molecular subtypes were defined as follows: Luminal A as (ER and/or PR+, HER2-, Ki67 < 15%); Luminal B (HER2-) as (ER and/or PR+, HER2-, Ki67 ≥ 15%); Luminal B (HER2+) as (ER and/or PR+, HER2+); HER2 type as (ER and PR-, HER2+);and TN as (ER, PR and HER2-).

### Statistical methods

We categorized the continuous or discrete birth size variables (birth weight, birth length, head circumference at birth) into approximately equally sized categories. We also examined the measures as quantitative increments of approximately one standard deviation (SD), i.e., 0.5 kg for birth weight, 2 cm for birth length, and 1.5 cm for head circumference.

We used Cox proportional hazards regression to study the association of the birth size indicators with risk of breast cancer subtypes, and precision of hazard ratios was estimated using 95% confidence intervals (CI). Breast cancer cases that could not be subtyped were censored at the date of diagnosis. To examine if the associations between birth size and breast cancer risk differed by breast cancer subtype, we used the data augmentation method described by Lunn and McNeil [22]. A small *p* value for heterogeneity from this test would indicate that the associations of birth size with breast cancer risk differs between tumour subtypes. Departure from the proportional hazards assumption was evaluated by Schönfelds residuals and by inspection of the log-log plots, and there were no clear violations to the assumption of proportionality. Attained age was used as the time scale in all analyses. In the multivariable analysis, we adjusted for gestational age, birth year in 5-year categories, and maternal birth order, age at birth, and socioeconomic status. In a subset of women (73%), we considered whether adjustment for maternal height and the adult risk factors age at first birth and parity influenced the findings.

In a separate analysis, we assessed whether the association of birth size with risk of luminal and non-luminal subtypes differed by maternal height. In that analysis, maternal height was dichotomized at the population median (163 cm). We used the likelihood-ratio (LR) test to assess this possible effect modification.

The statistical analyses were conducted using STATA version 13.1 (StataCorp).

## Results

Baseline characteristics of the cohort members according to birth size can be viewed in Table 1, and the distribution of breast cancer cases according to molecular subtype is shown in Table 2.

**Table 2:** Descriptive statistics for the 537 breast cancer cases with molecular subtyping

	Luminal A	Luminal B (HER-)	Luminal B (HER+)	HER 2 type	Triple negative	Total
Number (%)	232 (43.2)	175 (32.6)	65 (12.1)	24 (4.5)	41 (7.6)	537 (100)
Mean age at diagnosis (SD)	56.4 (9.8)	54.1 (11.3)	53.8 (9.8)	51.5 (10.7)	53.9 (11.3)	54.9 (10.5)
Tumour grade (%)						
1	58 (25.0)	3 (1.7)	1 (1.5)	1 (4.2)	0	63 (11.7)
2	155 (66.8)	93 (53.1)	19 (29.2)	3 (12.5)	6 (14.6)	276 (51.4)
3	19 (8.2)	79 (45.1)	45 (69.2)	20 (83.3)	35 (85.4)	198 (36.9)
Histopathological type						
Ductal	198 (85.3)	155 (88.6)	60 (92.3)	18 (75.0)	31 (75.6)	462 (86.0)
Lobular	24 (10.3)	8 (4.6)	2 (3.1)	0	0	34 (6.3)
Tubular	2 (0.9)	0	0	0	0	2 (0.4)
Mucinous	6 (2.6)	2 (1.1)	0	0	0	8 (1.5)
Medullary	0	6 (3.4)	3 (4.6)	6 (25.0)	6 (14.6)	21 (3.9)
Papillary	0	1 (0.6)	0	0	0	1 (0.2)
Metaplastic	0	0	0	0	2 (4.9)	2 (0.4)
Other	2 (0.9)	3 (1.7)	0	0	2 (4.9)	7 (1.3)
Tumour size, cm (%)						
< 2	151 (65.1)	79 (45.1)	26 (40.0)	4 (16.7)	14 (34.2)	274 (51.0)
2–5	49 (21.1)	59 (33.7)	28 (43.1)	10 (41.7)	16 (39.0)	162 (30.2)
> 5	5 (2.2)	7 (4.0)	1 (1.5)	0	2 (4.9)	15 (2.8)
Uncertain	27 (11.6)	30 (17.1)	10 (15.4)	10 (41.7)	9 (22.0)	86 (16.0)
Stage						
0	0	0	0	1 (4.2)	0	1 (0.2)
I	105 (45.3)	57 (32.6)	14 (21.5)	8 (33.3)	17 (41.5)	201 (37.4)
II	92 (39.7)	90 (51.4)	37 (56.9)	12 (50.0)	14 (34.2)	245 (45.6)
III	10 (4.3)	8 (4.6)	3 (4.6)	1 (4.2)	3 (7.3)	25 (4.7)
IV	4 (1.7)	4 (2.3)	4 (6.2)	1 (4.2)	1 (2.4)	12 (2.6)
Unknown	21 (9.1)	16 (9.1)	7 (10.8)	1 (4.2)	6 (14.6)	51 (9.5)

Table 3 shows the results for birth weight, birth length, and head circumference related to the risk for each molecular subtype. Birth length (per 2 cm increment) was positively associated with risk of Luminal A breast cancer (HR = 1.2, 95% CI, 1.0–1.3), Luminal B (HER2+) breast cancer (HR = 1.3, 95% CI, 1.0–1.7), and TN breast cancer (HR = 1.4, 95% CI, 1.0–1.9), but there were no clear associations with Luminal B (HER2-) and HER2 type (p for heterogeneity 0.8). For birth weight and head circumference, there were no clear associations with risk for any of the molecular subtypes.

**Table 3:** Indicators of birth size associated with risk of molecular subtypes of breast cancer

	Luminal A		Luminal B (HER2-)		Luminal B (HER2+)		HER2 type		Triple negative	
	***n***	HR (95% CI) ^a^	***n***	HR (95% CI)^a^	***n***	HR (95% CI)^a^	***N***	HR (95% CI)^a^	***n***	HR (95% CI) ^a^
Birth weight (g)										
< 3000	37	1.0 (0.6–1.5)	26	1.1 (0.7–1.8)	7	0.6 (0.2–1.4)	4	1.2 (0.3–4.1)	7	0.7 (0.3–2.0)
3000–3499	77	1.0	63	1.0	28	1.0	9	1.0	15	1.0
3500–3999	82	1.2 (0.9–1.7)	65	1.2 (0.8–1.7)	21	0.9 (0.5–1.6)	7	0.8 (0.3–2.2)	12	1.0 (0.5–2.2)
≥ 4000	31	1.3 (0.8–1.9)	16	0.8 (0.4–1.3)	9	1.1 (0.5–2.4)	4	1.1 (0.3–3.8)	5	1.2 (0.4–3.4)
Per 500 g increment	227	1.1 (1.0–1.3)	170	0.9 (0.8–1.1)	65	1.2 (0.9–1.6)	24	1.0 (0.6–1.4)	39	1.3 (0.9–1.8)
Birth length (cm)^b^										
< 49	35	1.0	23	1.0	6	1.0	5	1.0	5	1.0
49	20	0.6 (0–4-1.1)	16	0.7 (0.3–1.2)	11	1.8 (0.6–5.1)	2	0.4 (0.1–2.0)	6	2.0 (0.5–7.4)
50	60	1.1 (0.7–1.7)	46	1,0 (0.6–1.7)	14	1.3 (0.5–3.7)	7	0.7 (0.2–2.3)	9	1.8 (0.5–6.2)
51	35	0.9 (0.5–1.4)	37	1.2 (0.7–2.0)	10	1.3 (0.4–3.8)	5	0.8 (0.2–3.0)	7	2.1 (0.6–7.9)
> 51	77	1.2 (0.8–1.9)	49	1.0 (0.6–1.7)	24	2.0 (0.8–5.5)	5	0.5 (0.1–2.0)	12	2.4 (0.7–8.5)
Per 2 cm increment	227	1.2 (1.0–1.3)	171	1.0 (0.8–1.2)	65	1.3 (1.0–1-7)	24	0.8 (0.5–1.3)	39	1.4 (1.0–1.9)
< 50 cm	112	1.0	81	1.0	31	1.0	14	1.0	19	1.0
≥50	115	1.2 (0.9–1.6)	90	1.2 (0.9–1.7)	34	1.2 (0.7–2.0)	10	0.9 (0.4–2.2)	20	1.5 (0.8–3.1)
Head circumference (cm)										
< 34	40	1.0	26	1.0	10	1.0	5	1.0	9	1.0
34	50	1.0 (0.6–1.5)	39	1.0 (0.6–1.7)	16	1.2 (0.5–2.6)	7	0.9 (0.3–3.0)	7	0.7 (0.3–2.0)
35	59	1.0 (0.6–1.5)	50	1.1 (0.7–1.8)	19	1.2 (0.5–2.6)	6	0.6 (0.2–2.1)	10	0.9 (0.3–2.4)
36	46	1.1 (0.7–1.7)	34	1.1 (0.6–1.8)	11	1.0 (0.4–2.4)	5	0.8 (0.2–2.8)	7	1.0 (0.3–2.7)
≥ 37	31	1.3 (0.8–2.2)	22	1.3 (0.7–2.3)	8	1.4 (0.5–3.5)	1	0.3 (0.0–2.6)	6	1.5 (0.5–4.6)
Per 1.5 cm increment	226	1.1 (0.9–1.2)	171	1.0 (0.9–1.2)	64	1.1 (0.9–1.4)	24	0.9 (0.5–1.2)	39	1.1 (0.8–1.5)

^a^HRs adjusted for gestational age at birth, birth order, birth year in 5 year categories, maternal age at birth, and maternal socioeconomic status

^b^Lunn McNeil likelihood-ratio test, *p* = 0.7

The multivariable results did not substantially differ from those only adjusting for attained age. The results restricted to the subset of women with information on maternal height and the adult risk factors age at first birth and parity (results not tabulated) were similar to the main results, however, for some categories, the number of participants was low in the subset analysis.

In a separate analysis (Table 4), we assessed whether associations of birth length (per 2 cm increment) and molecular breast cancer subtypes, indicated as luminal or non-luminal breast cancer, differed by maternal height (dichotomized at the median, 163 cm). For non-luminal subtypes (HER2+ and TN), there was a positive association if mothers were 163 cm or taller (HR = 1.5, 95% CI, 1.0–2.2 per 2 cm), and no association if mothers were shorter than 163 cm (HR = 1.0, 95% CI, 0.7–1.6, per 2 cm, LR-test *p* = 0.4). For luminal breast cancer (Luminal A and Luminal B (HER2-)), there was a similar, but weaker pattern: thus, there was a weak positive association if mothers were 163 cm or taller (HR = 1.3, 95% CI, 1.1–1.5 per 2 cm) and no clear association (HR = 1.0, 95% CI, 0.9–1.2 per 2 cm) if maternal height was less than 163 cm (LR-test *p* = 0.7).

**Table 4:** Birth weight and length: associations with luminal and non-luminal breast cancer, by maternal height

	Luminal subtypes						Non-luminal subtypes					
	Maternal height < 163 cm			Maternal height ≥ 163 cm			Maternal height < 163 cm			Maternal height ≥ 163 cm		
	Cases	Incidence rate^**b**^	HR (95% CI)^**a**^	Cases	Incidence rate^**b**^	HR (95% CI)^**a**^	Cases	Incidence rate^**b**^	HR (95% CI)^**a**^	Cases	Incidence rate^**b**^	HR (95% CI)^**a**^
Birth weight (g)												
< 3000	32	37.2	1.0 (0.6–1-5)	24	35.2	1.0 (0.6–1.6)	6	7.0	1.0 (0.3–3.3)	3	4.4	0.5 (0.1–2.5)
3000–3499	82	42.5	1.0 (ref)	71	37.2	1.0 (ref)	10	5.2	1.0 (ref)	10	5.2	1.0 (ref)
3500–3999	81	56.5	1.3 (1.0–1.8)	74	36.7	1.0 (0.7–1.4)	4	2.8	0.5 (0.2–1.7)	11	5.5	1.1 (0.5–2.7)
≥ 4000	21	45.2	1.0 (0.6–1.6)	33	38.3	1.1 (0.7–1.7)	3	6.5	1.1 (0.3–4.2)	5	5.8	1.3 (0.4–3.8)
Per 500 g	216	46.0	1.1 (0.9–1.3)	202	36.9	1.1 (0.9–1.3)	23	4.9	0.7 (0.7–1.6)	29	5.3	1.3 (0.9–1.9)
Birth length (cm)^c,d^												
< 49	33	43.9	1.0 (ref)	17	31.2	1.0 (ref)	6	8.0	1.0 (ref)	2	3.7	1.0
49	29	40.5	0.8 (0.5–1.4)	14	23.8	0.8 (0.4–1.6)	4	5.6	0.9 (0.2–3.5)	2	3.4	1.9 (0.2–11.0)
50	63	54.8	1.1 (0.7–1.6)	40	34.9	1.1 (0.6–2.1)	7	6.1	0.9 (0.3–3.1)	5	4.4	2.4 (0.3–18.0)
51	35	40.4	0.9 (0.5–1.4)	41	37.2	1.3 (0.7–2.5)	3	3.5	0.7 (0.1–3.0)	6	5.5	3.5 (0.5–26.3)
≥ 52	56	46.4	1.0 (0.6–1.6)	90	43.1	1.6 (0.9–2.9)	3	2.5	0.5 (0.1–2.5)	14	6.7	4.4 (0.6–30.6)
Per 2 cm	216	46.0	1.0 (0.9–1.2)	202	36.9	1.3 (1.1–1.5)	23	4.9	1.0 (0.7–1.6)	29	5.3	1.5 (1.0–2.2)

^a^HRs adjusted for gestational age at birth, birth order, birth year in 5 year categories, maternal age at birth, and maternal socioeconomic status

^b^Per 100,000 person years

^c^Likelihood-ratio test for the association of birth length with luminal subtypes of breast cancer according to maternal height, *p* = 0.7

^d^Likelihood-ratio test for the association of birth length with non-luminal subtypes of breast cancer according to maternal height, *p* = 0.4

## Discussion

In this long-term follow-up for breast cancer, we found that birth length was positively associated with risk both for Luminal A, Luminal B (HER2+), and TN breast cancer, and the results suggest that these associations displayed a homogeneous pattern, based on the test for heterogeneity. We also found evidence that the positive association of birth length was present in women whose mothers were relatively tall (at population median or taller).

To understand these findings, the literature does not provide any direct help, because risk of molecular breast cancer has not been previously studied in the context of birth size. However, one study has assessed birth weight with risk for breast cancer according to hormone receptor status.

[10], and found that a positive association with risk for ER positive, but not for ER negative breast cancer. Although that study used birth weight as indicator for birth size, and our results suggested a positive association limited to birth length, the luminal subtypes of breast cancer overlap to a substantial degree with ER positive tumours, and the results may therefore support each other.

However, the positive association that we found for birth length was also present for the HER+ subtype and for triple negative (TN) breast cancer. Moreover, the strength of the positive associations was rather similar for the different subtypes.

The major strength of the present study is the large cohort of women born between 1920 and 1966, and the long-term breast cancer follow-up provided by record linkage with the Cancer Registry of Norway. The notification of cancer to the Cancer Registry of Norway is mandatory and regulated by law, and the registration is virtually complete [23]. Baseline information on birth size was measured and stored in birth records that we have transcribed and computerized, and later combined with adult information about age at first birth and parity. We used archival diagnostic tumour tissue to determine the molecular subtype, using a predetermined subtyping algorithm [3, 4], and the same antibodies and cut-off levels for all cases. However, in the present study, we classified the Basal phenotype and the Five negative phenotype as TN in order to avoid subtypes with relatively few cases.

Limitations of the study include lack of longitudinal information on childhood and adolescent growth, including height and weight gain, which could be intermediate factors, and have been shown to be associated with breast cancer risk in previous studies [24, 25]. For ethical reasons, we were not allowed to contact cohort members who were still alive to update relevant information. Another limitation of our study is the lack of biological material from the time of birth. Possibly, measurements of cord blood hormones would be a useful approach to address the intrauterine roots of breast cancer more directly [26], but because participants were born between 1920 and 1966, biological material from their births could not be expected to be available. Nonetheless, measures of birth size (birth weight, birth length, head circumference) could be interpreted as proxy factors for intrauterine growth and hormonal exposures in utero [26, 27]. Lastly, there is always a concern linked to the representativeness of cases in a single observational study, and therefore, our results need verification in other studies.

The main finding of this study suggests that birth length is positively associated with risk of Luminal A and Luminal B (HER2+) breast cancer, both of which are ER positive tumours, and with TN breast cancer, which is ER negative. Interestingly, associations of birth length were not consistent across ER and HER2 status, as indicated by the lack of association for the HER2 type, which is ER negative, and the Luminal B (HER2-) type, which is ER positive. This suggests that the association of birth length with breast cancer risk is still unresolved and may involve other factors than ER and HER2. Despite the relatively large study cohort, the number of incident cases was still moderate, and the results of a larger study, or from other cohort studies, would most likely contribute with useful insights that would nuance our findings.

## Conclusions

The fact that birth length was the only measure of birth size that was associated with any of the subtypes, may point to longitudinal growth as particularly important, regardless of ER status. It is also compelling that the positive association of birth length was present only among women whose mothers were relatively tall. This may further support the possibility that determinants of longitudinal growth may be at the core of the intrauterine roots of breast cancer [11, 28].

## Data Availability

The data sets generated and analysed during the current study are not publicly available due to privacy reasons, but anonymized data may be available from the corresponding author on reasonable request and after approval of the regional ethical committee.

## Abbreviations

CI: Confidence interval
ER: Oestrogen receptor
HER2: Human epidermal growth factor receptor 2
HR: Hazard ratio
PR: Progesterone receptor
